# Apigenin and Abivertinib, a novel BTK inhibitor synergize to inhibit diffuse large B-cell lymphoma* in vivo and vitro*

**DOI:** 10.7150/jca.34981

**Published:** 2020-02-03

**Authors:** Shujuan Huang, Mengxia Yu, Nana Shi, Yile Zhou, Fengling Li, Xia Li, Xin Huang, Jie Jin

**Affiliations:** 1 Department of Hematology, the First Affiliated Hospital, Zhejiang University College of Medicine, Hangzhou, People's Republic of China; 2 Key Laboratory of Hematologic Malignancies, Diagnosis and Treatment, Zhejiang, Hangzhou, People's Republic of China; 3 Department of Hematology, Hangzhou First people's hospital, Zhejiang, Hangzhou, China; 4 The Children's Hospital Zhejiang University School of Medicine

## Abstract

**Background**: Apigenin, a flavonoid phytochemical extracted from fruits and vegetables, has shown anti-neoplastic effects in a variety of malignant tumors. DLBCL is the most common type of aggressive lymphoma in adults with a poor prognosis. Small-molecule inhibitors like BTK inhibitors have demonstrated extended period of disease control. Whereas the effects of the synergetic inhibition of the two have not been elucidated.

**Methods**: We assessed the efficacy of Apigenin alone or combined with Abivertinib to inhibit DLBCL progression. Cell viability was examined using the cell proliferation cell proliferation assay (MTS). Apoptotic cells and cell cycle evaluation were detected by Annexin V-FITC and DNA staining solution respectively. Western blot was used to explore the potential mechanism, and the* in vivo* effects of the two drugs were performed by a DLBCL xenograft BALB/c nude mice model.

**Results**: Our results demonstrated that Apigenin can inhibit the proliferation and clone forming of DLBCL cells. Apigenin also induces apoptosis by down-regulating BCL-XL and activating Caspase family. In addition, Apigenin down-regulates cell cycle proteins including CDK2/CDK4/CDK6/CDC2/p-RB to increase G2/M phase arrest. Mechanically, our data demonstrate that Apigenin leads to a significant reduction of the expression of pro-proliferative pathway PI3K/mTOR to inhibit DLBCL cells survival. Moreover, our* in vitro* and *in vivo* results show that Apigenin can synergize with Abivertinib, a novel BTK inhibitor, in treating DLBCL visa synergistically inducing apoptosis and inhibiting the p-GS3K-β and its downstream targets.

**Conclusions**: Collectively, our study suggests that Apigenin exerts improving anti-lymphoma effect of BTK inhibitors and provides hope to targeted therapy of those develop resistance.

## Introduction

Aggressive B-cell lymphomas cause significant mortality and morbidity worldwide, mainly due to drug insensitivity or therapeutic resistance 1, 2. DLBCL is the most common type of aggressive lymphoma in adults and accounts for 30% of lymphomas and 40% of non-Hodgkin lymphomas (NHL). As for therapy, standard treatment is R-CHOP (rituximab, cyclophosphamide, doxorubicin, and vincristine, prednisone) chemo-immunotherapy, resulting in about 50-60% achieving a durable complete response while 30-40% of patients fail to react to upfront therapy and remain refractory or relapse (R/R) 3. Small-molecule inhibitors (SMIs) are a promising class of treatments for patients with chemo-refractory DLBCL. Several studies with these agents have convincingly demonstrated extended period of disease control in responding patients without meaningful toxicity. Bruton's tyrosine kinase (BTK) is a non-receptor kinase that plays an oncogenic role in the proliferation and survival of many B cell malignancies. More recently, small-molecule inhibitors of this kinase have shown excellent anti-tumor activity, especially in patients with relapsed/ refractory chronic lymphocytic leukemia (CLL) and mantle-cell lymphoma (MCL). Our earlier studies also demonstrated that the novel BTK inhibitor Abivertinib is effective in inhibiting MCL survival 4. Efficacy of BTK inhibition as a single agent therapy is powerful, but resistance may develop, fueling the development of combination therapies that improve clinical responses 5-13. Recent studies have investigated the combination of BTK inhibitor PLS-123 and the mammalian target of rapamycin (mTOR) inhibitor everolimus synergy to attenuate proliferation and motility of MCL cell lines 13.

Results from multiple case-control studies indicate that High intakes of fruit and vegetables may reduce the risk of cancer 14-21. Here we propose Apigenin, a bioflavonoid extracted from plants such as fruits and vegetables. Over the last years, a considerable number of *in vitro* and *in vivo* studies affirmed the anti-tumor effect and safety of Apigenin including prostate cancer, breast cancer, thyroid cancer, colorectal cancer, bladder cancer, skin cancer, bone cancer and leukemia 22-29. However, to our knowledge there was no research to discuss the effects of Apigenin in DLBCL and their underlying mechanisms.

In this study, our results demonstrated Apigenin can inhibit the DLBCL progression and can cooperate with Abivertinib to achieve better anti-lymphoma function *in vivo and vitro*.

## Methods and Materials

### Materials

Abivertinib (AC0010) was kindly provided by ACEA Pharmaceutical Research (Hangzhou, China), Apigenin was purchased from Selleckchem Company (Houston, USA). Abivertinib and Apigenin were dissolved in dimethyl sulfoxide (DMSO; Sigma-Aldrich, St. Louis, MO, USA) and sub-packaged in different concentration stored at -20°C. Rabbit polyclonal antibodies to BTK, p-BTK(Tyr223), PLCγ2, p-PLCγ2(Tyr1217), p-IKKα/β(Ser176/180), NF-κB, p-NF-κB, ERK (MAPK), p-ERK (p-MAPK) (Thr202/Tyr204), PI3K, AKT, p-AKT(Ser473), GSK3β,p-GSK3β (Ser9), Bcl-2, Bax, Bcl-xl, Bim, Bad, MCL-1, caspase-3, 7, 8 and PARP were from Cell Signaling Technology (Beverly, MA, USA).

### Cell cultures

The DLBCL cell lines U2932, OCI-LY10 were kindly donated by ACEA Pharmaceutical Research (Hangzhou, China). These two cell lines were cultured in RPMI-1640 (Gibco, Billings, MT, USA) supplemented with 10% fetal bovine serum (FBS, Gibco, Billings, MT, USA), 100 μc/ml penicillin, and 100 U/ml streptomycin.

### Growth inhibition assay

DLBCL cells were seeded in 96-well culture plates at proper density (1 × 10^5^ viable cell-line cells). Cells were treated with ABIVERTINIB and Apigenin of different concentrations, respectively, for 24 h and (or) 48 h; at the same time, equal volume of DMSO was added to cells as negative control. Colorimetric CellTiter 96 Aqueous One Solution Cell Proliferation Assay (MTS assay, Promega, Madison, WI, USA) was used to determine the cytotoxicity. The absorbance at 490 nm was measured for each well. IC50 was calculated by CalcuSyn Software (Biosoft, Cambridge, UK). Cell-line experiments were triplicated.

### Colony forming Assay

U2932 cells were treated with different drugs (Apigenin2.5μM, AC0010 0.156μM,combination)for 14 days, and colonies were stained with 0.05% crystal violet solution for 30 min then counted.

### Detection of apoptosis

Refer to Annexin V/PI Apoptosis Detection Kit (BD Pharmingen, San Diego, CA, USA) for operation. Cells were harvested after exposure to various drugs of diverse doses or equal volumes of DMSO for 24 h. About 5×105 cells were collected from each sample, washed three times with1 × PBS, and resuspended in binding buffer (10mM HEPES/NaOH [pH7.4], 140mM NaCl, 2.5mM CaCl2). 5μL Annexin V-FITC and 10 μL PI were added into the cell suspension. The mixture should be incubated in dark at room temperature for 10 min before using a flow cytometry (Becton Dickinson, Franklin Lakes, NJ, USA) to detect apoptotic cells.

### Evaluation of cell cycle

Refer to the Cell Cycle Detection Kit operation. Cells were harvested after exposure to ABIVERTINIB or Apigenin of diverse doses or equal volumes of DMSO for 24 h, About 1×106 cells were collected from each sample, washed three times with 1×PBS, and fixed with 75% ethanol at 4℃overnight. These fixed cells were suspended in DNA staining solution; after incubating in dark at room temperature for 30 min, the results were analyzed by flow cytometry (Becton Dickinson, Franklin Lakes, NJ, and USA).

### Western blot analysis

Cells from various conditions were harvested and washed twice in PBS and lysed a RIPA buffer (Cell Signaling Technology, Beverly, MA, USA) on ice for 30 min. Cells were then centrifuged at 12000g for 15 min at 4℃ and the supernatant was collected. The protein concentration was determined using BCA reagent. Protein samples were separated by 10% SDS-PAGE gel (Life Technology, USA) and transferred to a PVDF membrane (Millipore, Billerica, MA, USA). Next, the membranes were blocked in Tris-buffered solution (TBS) containing 5% non-fat milk for 1h and incubated with primary antibodies overnight at 4℃. After washed with TBS-T buffer three times, membranes were incubated with secondary antibodies (CST, Beverly, MA, USA) for 1h. The target protein bands were visualized using an ECL kit (Amersham, Little Chalfont, and UK) and analysis by the image lab software (bio-rad, california, USA).

### DLBCL xenograft model

Twenty-four male BALB/c nude mice (4 weeks of age) were obtained from the SHANGHAI SLAC LABORATORY ANIMAL CO. LTD and housed in Laboratory Animal center of Zhejiang Academy of Medical Sciences (Zhejiang, China. All mice were fed commercial diet and water ad libitum. U2932 cells were resuspended in 1×PBS. Cell suspension (1×10^7^ cells) was subcutaneously injected into the upper border of the groin of mice and incubated for 14 days to form xenofraft tumors when it reached to about 100 mm^3^. Animals with tumors were randomly assigned to one of four treatment groups (vehicle, 2 mg/kg Apigenin, 30mg/kg ABIVERTINIB, and combination every 2 days). At the end of the study for 10 days administration, animals were sacrificed and body weight was determined. Tumors were removed, measured, and weighed individually. Our animal study was approved by Ethics Committee for Laboratory Animals of the First Affiliated Hospital, College of Medicine, Zhejiang University (Hangzhou, China) and were conducted in accordance with the National Institutes of Health Guide for the Care and Use of Laboratory Animals.

### Statistical analyses

Mann-Whitney test was performed to assess statistical significance from treated sample compared to controls. Results with *p* < 0.05 were considered statistically significant (*). Results represent the median and in some instances mean ± SD of 3 independent experiments. For Western blotting, data were representative images of 3 independent experiments. For animal experience, weight change and average survival days were used to mean the mean or minus standard error (SEM).

All methods were performed in accordance with the relevant guidelines and regulations.

## Results

### Apigenin inhibits proliferation and cloning forming of diffuse large B-cell lymphoma cells

**Figure 1:**
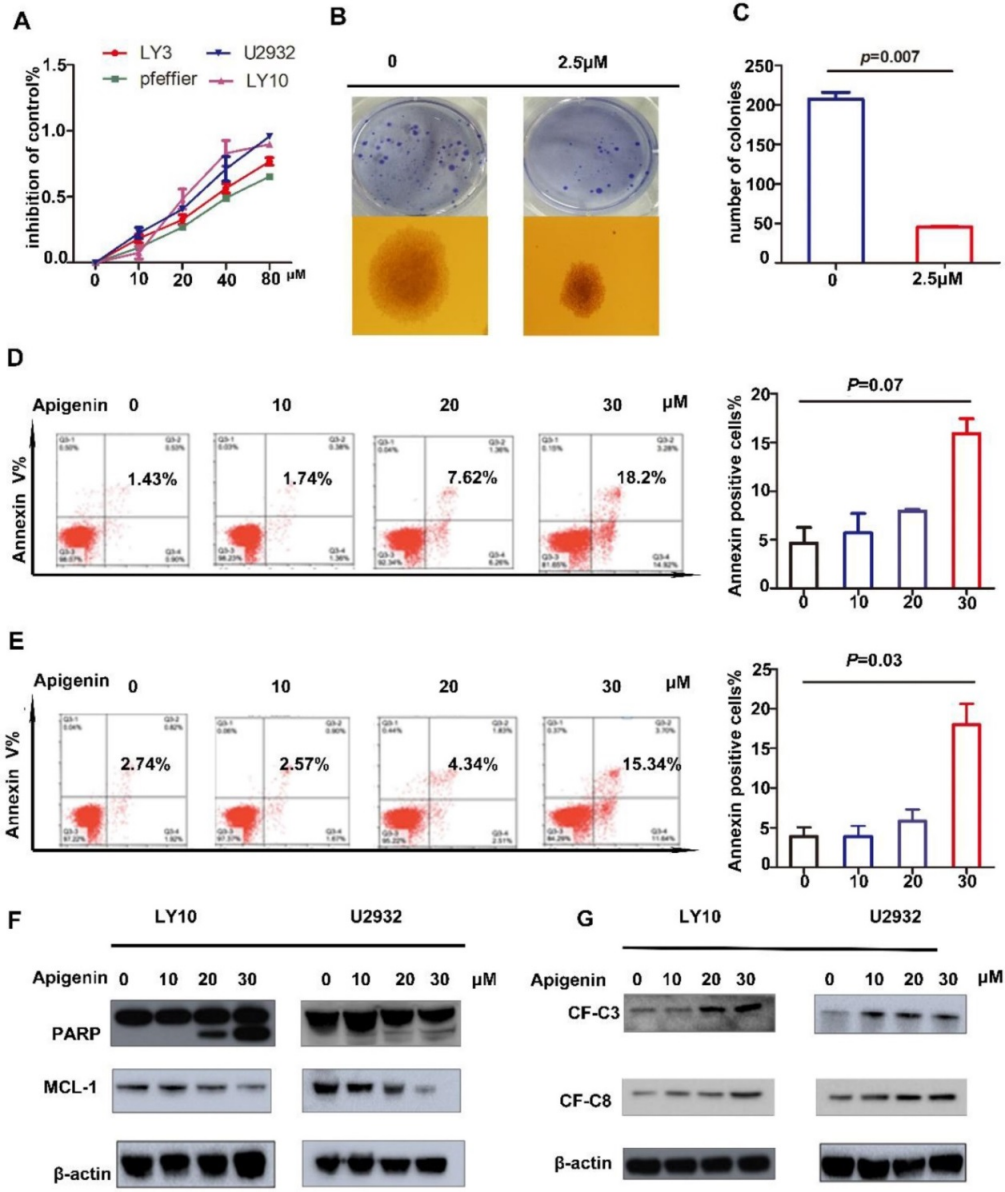
** (A)** DLBCL cell lines were treated with increasing doses of Apigenin (Api) (10-80µM) for 24 hours and the inhibition of cell proliferation was assessed (MTS assay). Data were normalized to DMSO-treated cells and represent the mean (SD) of 3 experiments. **(B)** And **(C)** Colony-forming assays in DLBCL cell line, U2932 was performed to show the number of colonies or colony-forming cells(1X). Data were normalized to DMSO-treated cells (*p=*0.007). **(D)**LY10 and **(E)**U2932 were treated with increasing doses of Apigenin for 24 h, cells were co-stained with Annexin V and PI and apoptosis was measured by FCM(*p=*0.07 and *p=*0.03). Expression of **(F)** PARP,MCL-1 and **(G)** cleaved-caspase3 and cleaved-caspase-8 were analyzed by Western blotting analyses in cell lines referred above after treated with increasing dose of Apigenin 24 h.

Studies have shown that Apigenin has an anti-tumor effect in solid tumors as well as MM and AML cell lines. In this study, to explore the role of Apigenin in diffuse large B-cell lymphoma, we expose four representative DLBCL cell lines to increasing dose of the drug for 24 hours and measure cell viability. The results show that Apigenin inhibits the proliferation of all four cell lines with a dose dependent manner (Fig. 1A). Meanwhile, in order to confirm the cloning forming of DLBCL cells affected by Apigenin, we performed a soft agar colony formation test, which showed that the flavonoid can inhibit the clone formation of U2932 at a very low concentration about 2.5μM after two weeks' incubation (Fig. 1B and C).

### Apigenin induces apoptosis in diffuse large B-cell lymphoma cells

There has already studies showed that Apigenin could induce apoptosis in HL60 and other cells. In order to study whether Apigenin can induce apoptosis in DLBCL, we played apoptosis assay with Annexin V-FITC and PI double staining followed by flow cytometry. The results demonstrate that significant of apoptosis induced by Apigenin at a concentration about 20μM in U2932 and LY10 (Fig. 1D). Next, to further reveal the mechanism of Apigenin-induced apoptosis, we perform a screen assay about proteins associated with mitochondrial apoptosis signal pathway and the downregulation of anti-apoptotic proteins MCL-1 was detected (Fig. 1F). However there was no significant change in BCL-2 and BCL-XL. Meanwhile, the results demonstrate caspase family proteins, we detected the expression of cleaved-PARP, cleaved-C3 and cleaved-C8, respectively with a dose-dependent manner (Fig. 1G). We claim that Apigenin can influence both mitochondria and caspase family apoptotic pathways to induce apoptosis in diffuse large B cells.

### Apigenin disturbs the cell cycle of diffuse large B-cell lymphoma

**Figure 2:**
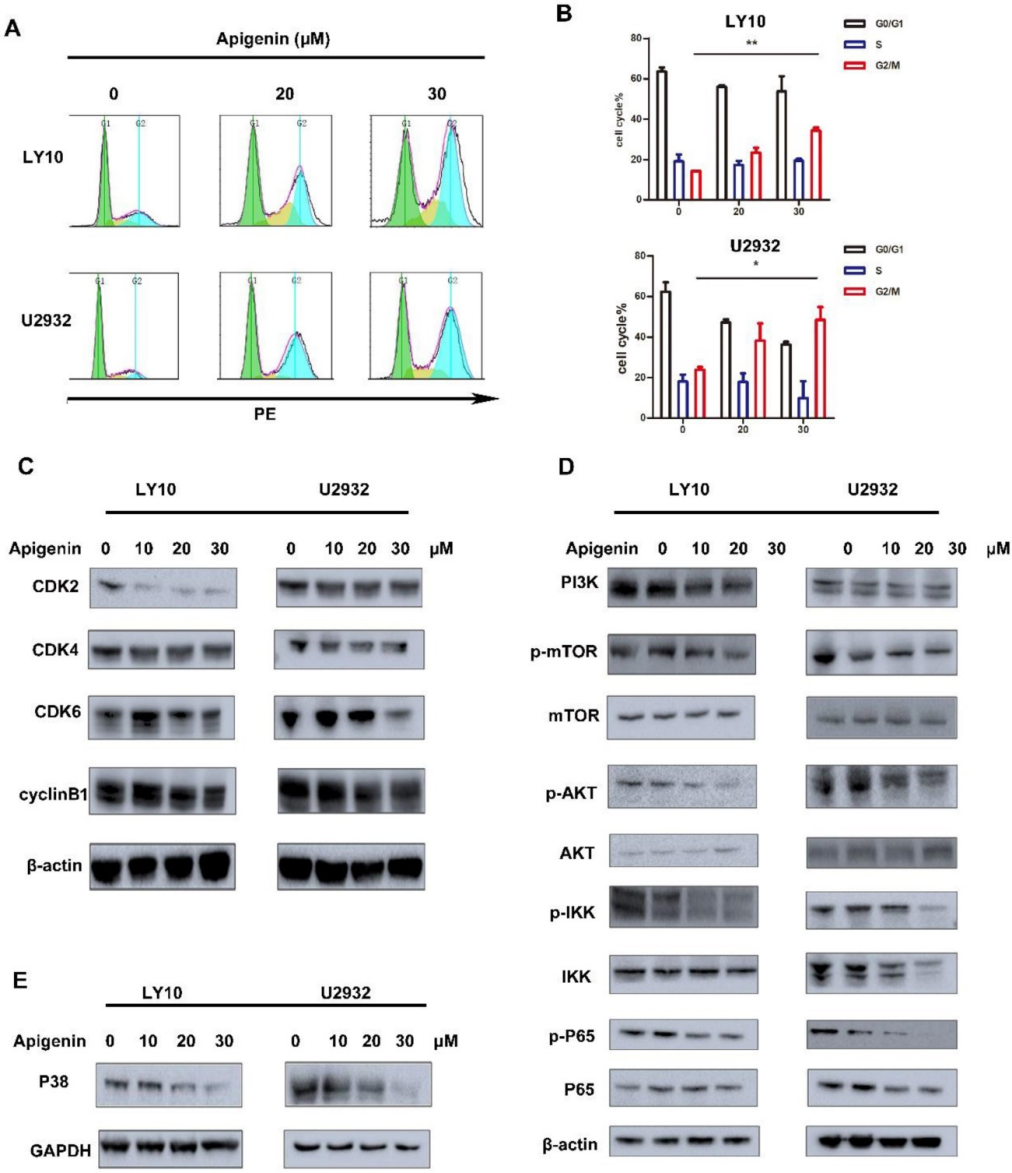
** (A)** and **(B)** DLBCL cell lines (LY10/U2932) were treated with increasing doses Apigenin for 24 h. The cells were stained with propidium iodide and underwent FCM analysis to determine cell cycle distribution. **(C)** Soluble proteins CDK4, CDK6, CDK2, Rb and cyclinB of LY10 and U2932 were analyzed by Western blotting analyses at the indicated concentrations for 24 h. **(D)** DLBCL cell lines were treated with increasing dose Apigenin for 24 h. Western blot analysis was conducted for PI3K and its downstream pathway. **(E)** DLBCL cell lines were treated with increasing dose Apigenin for 24 h. Western blot analysis was conducted for MAPK.

Earlier, it was reported in the literature that Apigenin can induce cell cycle arrest in leukemia cell lines, such as G2/M phase arrest in HL60 and G0/G1 phase block in TF-1. In our study, we demonstrated that Apigenin disturbs the cell cycle in diffuse large B cells. Analysis from the results of flow cytometry show that Apigenin can induce significant G2/M phase arrest in U2932 and LY10 at a very low dose(Fig. 2A and B), so it may be the main mechanism of effect of Apigenin in diffuse large B-cell lymphoma. Mechanismly study of the performance, we give the results about downregulation of CDK2, CDK4, CDK6 and Rb, CDC (Fig. 2C), which is consistent with flow results above.

### Apigenin alters PI3K/mTOR/AKT and MAPK pathway

The activation of PI3K signal pathway play a key role in the survival of multiple tumors. In our study, we show that Apigenin can effectively inhibit the activation of PI3K and proteins like p-mTOR, p-AKT, p-IKK, p-p65 involved in PI3K signal pathway (Fig. 2D), thereby effectively achieve the anti-tumor effects. Meanwhile, we found that Apigenin can also down-regulate the important protein P38 in MAPK pathway and thus play an anti-tumor effect (Fig. 2E).

### Apigenin synergizes with Abivertinib to inhibit proliferation and colony formation of diffuse large B-cell lymphoma

**Figure 3:**
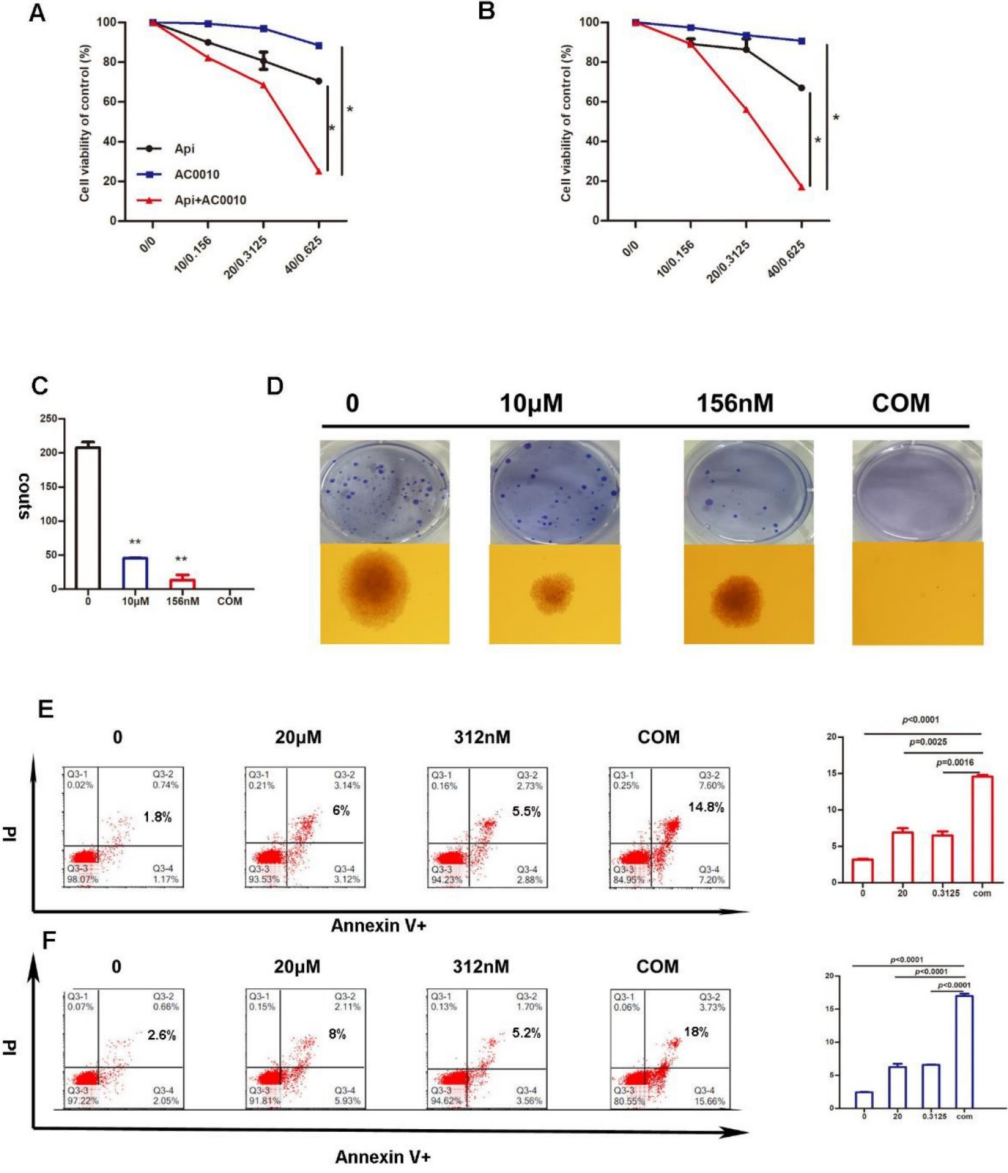
Inhibition in cell proliferation induced by Apigenin, abivertinib, and combination in LY10 **(A)** and U2932 **(B)** cells after incubation of 24 hours. **(C)** And **(D)** Colony-forming assays in DLBCL cell line, U2932 was performed as above and show the number of colonies. Data were normalized to DMSO-treated cells. Apoptosis induced by Apigenin, abivertinib, and Apigenin plus abivertinib after 24h in LY10 **(E)** and U2932 **(F)** cell lines.

In this study, to determine whether BTK inhibitor-based drug combination therapy can benefit the treatment of diffuse large B-cell lymphoma, we play proliferation assay with an appropriate concentration of Apigenin in combination with low-dose Abivertinib in U2932 and OCI-LY10 for 48 hours. Our results indicate that the cell viability of the combined group is significantly lower than either of the single drug groups (Fig. 3A, B). Next, the results of soft agar colony formation experiments further confirmed that the colony forming ability of the combined group in U2932 is sufficiently inhibited (Fig. 3D).

### Apigenin synergizes with Abivertinib to induce apoptosis of diffuse large B-cell lymphoma

**Figure 4:**
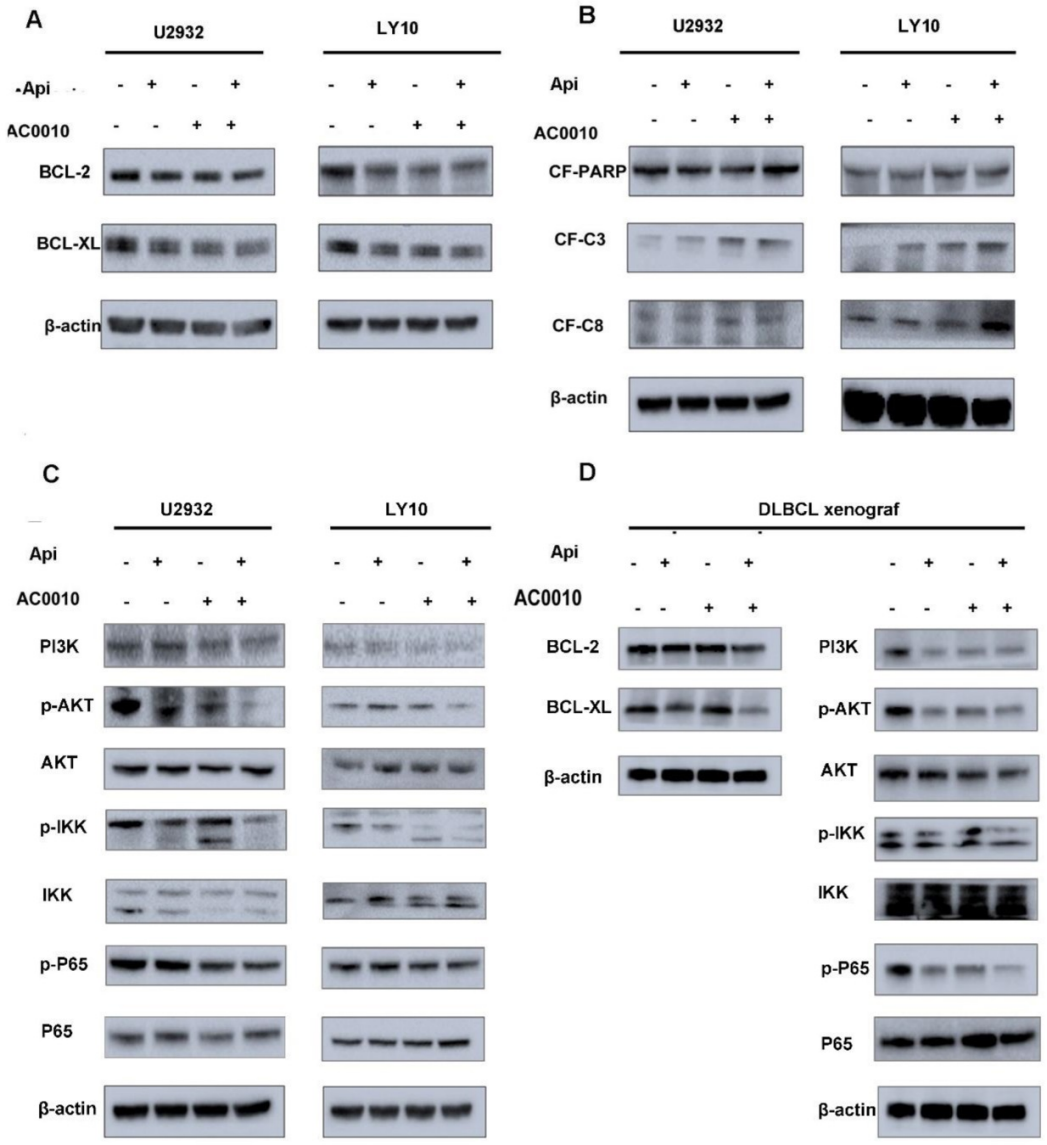
DLBCL cell lines were treated with Apigenin, abivertinib, and Apigenin plus abivertinib for 24 h respectively. Western blot analysis was conducted for **(A)** BCL2, BCL-XL **(B)** cleaved-PARP, cleaved-caspase3, cleaved-caspase8. **(C)** U2932 and LY10 cells were treated with Apigenin, abivertinib, and Apigenin plus abivertinib for 24 h, Western blot analysis was conducted for PI3K, p-AKT, p-IKK, p-P65.** (D)** Tumor tissue was removed from the DLBCL xenograft and total protein was extracted. Western blot analysis was conducted for BCL-2, BCL-XL, PI3K, p-AKT, p-IKK, p-P65.

In order to further explore the mechanism of the combination effect, we firstly detect apoptosis in combination and single treatment groups. The results are shown in Fig. 3E and 3F: the combination of two drugs at a lower concentration can significantly induce apoptosis and is more than any single drug group. In the search for apoptotic mechanisms, we conclude that the combination of two drugs can down-regulate the anti-apoptotic proteins of BCL2 and BCL-XL (Fig. 4A), while the cleaved-PARP, cleaved-C3, cleaved-C8 were also captured (Fig. 4B). Meanwhile, we studied the expression level of BCL-2 and BCL-XL of different treatment group by Westernblot assay of tumor tissue moved from DLBCL xenograft and turned out the similar result to cell lines (Fig. 4D). According to our results, it was concluded that Apigenin synergizes with Abivertinib to induce apoptosis in diffuse large B-cell lymphoma by down-regulating BCL2, BCL-XL and activating the caspase family.

### Apigenin synergizes with Abivertinib to inhibit the proliferation of diffuse large B-cell lymphoma via down-regulating PI3K/p-AKT/p-IKK/p-P65 signal pathway

In our previous studies (data has not been published), we found that Abivertinib can inhibit multiple signaling pathways to achieve its effects. We used Western blotting to detect changes in the signaling pathways when Apigenin combined with Abivertinib in U2932 and OCI-LY10. The Abivertinib-targeted BTK and its downstream PLC-γ2 did not have a significant combined effect, and neither the STAT family proteins (data were not shown). Interestingly, we found that PI3K/p-AKT/p-IKK/p-P65 axis was significantly downregulated in the drug combination group (Fig. 4C). In the meantime, we explored the level of these proteins in the different treatment group of DLBCL xenograft (Fig.4D). The result *in vivo* were consistent with our *in vitro* data which demonstrated the combination of the two drugs can significantly downregulated PI3K and its downstream signal, thus inhibiting the abnormal proliferation of diffuse large B cells.

### Apigenin synergizes with Abivertinib inhibit the progression *in vivo* DLBCL models

**Figure 5:**
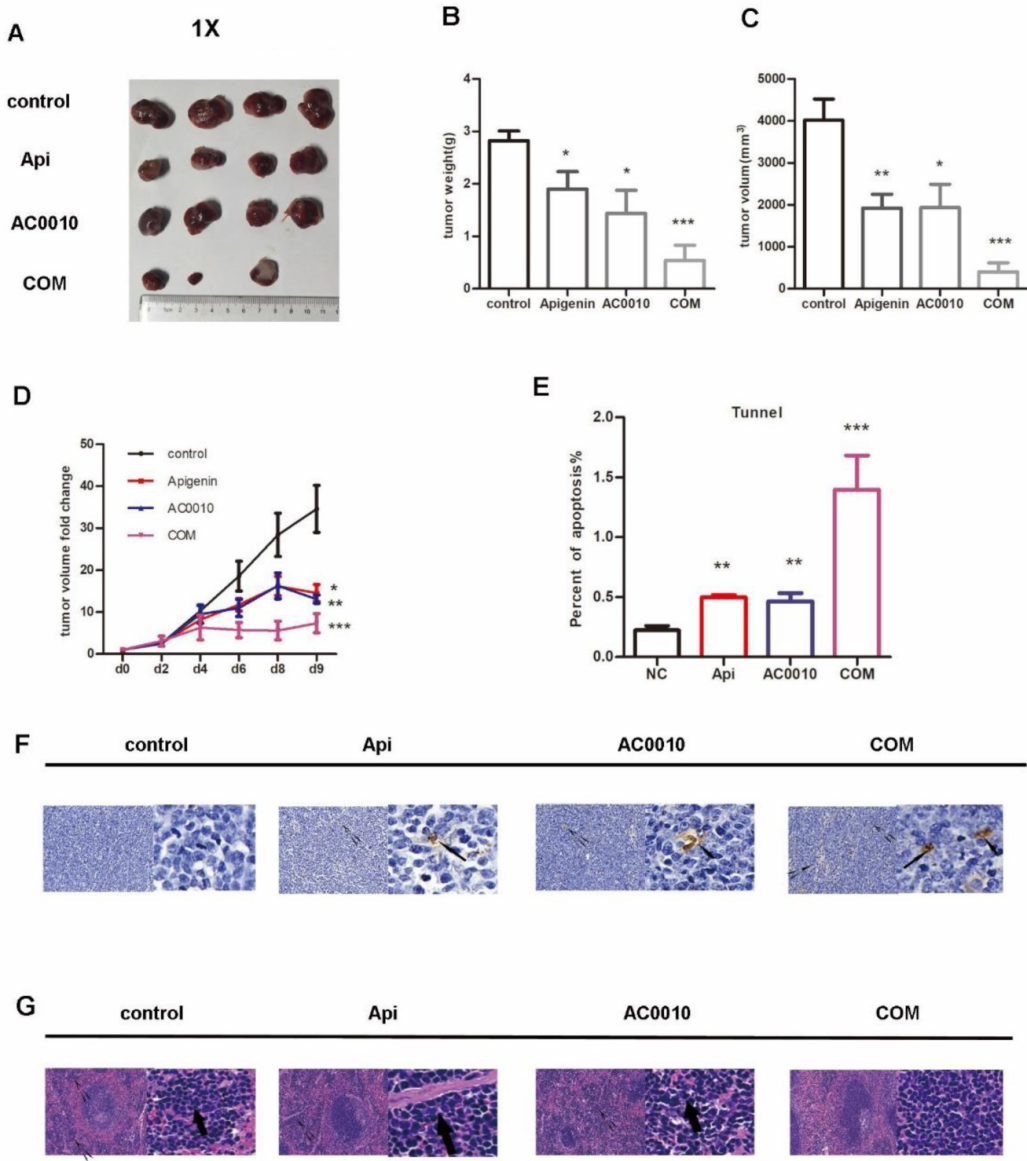
** (A)** Representative tumors in the xenograft mice treated in different groups, the treatments of Apigenin and Abivertinib or their combination significantly reduced tumor mass compared to vehicle. **(B)**Tumor weight in the group of Apigenin, Abivertinib, combination decreased compared to vehicle group. **(C)**Tumor size in the group of Apigenin, Abivertinib, combination decreased compared to vehicle group. **(D)** Tumor progression analyzed by measuring the size of tumors in each group every 2 days, the treatments effectively inhibit the growing of tumors. **(E)** And **(F)** Tunnel assay showed increase apoptosis in treatment group especially combination group (20X and 50X). **(G)** HE staining of spleen showed decreasing extramedullary hematopoiesis in combination group (20X and 50X).

All mice were treated as described in methods. Representative tumors in the xenograft mice treated in different groups are shown in Fig. 5A, the treatments of Apigenin and Abivertinib or their combination significantly reduced tumor mass compared to vehicle. In details, Apigenin, Abivertinib, COM decreased by 32.5%, 48%, 80% the tumor weight compared to vehicle group respectively (Fig. 5B).The similar results were found when refer to tumor size(Fig. 5C). Next, we analyzed the tumor progression by measuring the size of tumors in each group every 2 days, the result shown in Fig. 5D, the treatments effectively inhibit the growing of tumors. In order to measure the apoptosis induced by drugs, we played tunnel assay, the results showed increase in combination group (Fig. 5E and F).When analyze the tumor burden on spleen and liver of mice, the HE staining showed no significant difference among groups on liver while decreasing extramedullary hematopoiesis in combination group (Fig. 5G). All our results unveiled the fact that Apigenin can inhibit the DLBCL progression and can cooperate with Abivertinib to achieve better anti-lymphoma function.

## Discussion

In the present study, we demonstrate Apigenin decreases the proliferation and clones forming of DLBCL cells, induces apoptosis, disrupts cell cycle, and down-regulated PI3K and MAPK signal pathways in DLBCL. In the following research, we combined Apigenin and Abivertinib for the first time and achieved good combined effects* in vitro* and *in vivo*.

DLBCL is an aggressive and incurable malignant disease. Despite of standard chemotherapy, relapse and mortality are common, making the need for the development of novel targeted drugs or combination of therapeutic regimens. More recently, small-molecule inhibitors are a promising class of therapeutics for patients with chemo-refractory DLBCL such as the oncogenic protein BCL-2 and BTK^6^, ^11^. It has been reported in a recent study that the combination of ibrutinib and venetoclax benefit patients with mantle-cell lymphoma who had been predicted to have poor outcomes with current therapy 30.

In this study, we propose Apigenin which has been used as a dietary supplement due to its antitumor properties as a candidate agent to be combined with a novel BTK inhibitor Abivertinib 31. In the present study, we demonstrated the anti-DLBCL activity of Apigenin both in *in vitro* and *in vivo*, and found that Apigenin treatment inhibited the proliferation of DLBCL cell lines and process of U2932-mice models. The growth inhibition caused by Apigenin occurred in a dose-dependent manner. Our findings were in agreement with studies of other cancer types. Also in our results demonstrated that the inhibitory effect of Apigenin is associated with the increase of cell death. Treatment with Apigenin significantly inhibited the cell proliferation in U2932 and LY10 cells and resulted in apoptosis, as evidenced by the increased number of apoptotic cells and the activation of caspase and BCL2 family members. Meanwhile, Apigenin induced cytotoxic effects through causing cell cycle arrest at G2/M period as a result of the down-regulation of CDK2, CDK4, CDK6, Rb and CDC2 expression.

The activation of PI3K signal pathway plays a key role in the survival of multiple tumors and there have already been various inhibitors, such as (PI3Ki), exert a potent anti-tumor effect with or without a problem of off-target. In the further study, we next demonstrated Apigenin inhibited the activation of PI3K and MAPK pathway in DBLBL. Taken together, the results suggested that Apigenin inhibits the proliferation of DBLBL cells by up-regulating the cell apoptosis rate, disturbance of cell cycle and down-regulation of pro-proliferation pathway.

The combination chemotherapy regimen based on CD20 monoclonal antibody is widely applied in B-cell lymphoma and turned a pretty good outcome. The BTK inhibitor ibrutinib has been recognized in the treatment of B cell malignant hematological tumors. We hope for better therapy effect through combining Abivertinib, a novel BTK inhibitor, and Apigenin. Interestingly, when Apigenin combined with Abivertinib, it achieved much better inhibitory effect in DBLBL cells. In the present study, our results demonstrated Apigenin synergized with Abivertinib to induce apoptosis and inhibition of PI3K/p-AKT/p-IKK/p-P65 activation *in vivo and vitro*.

There has already been studies about Apigenin for cancer therapy* in vitro and in vivo* in the past several years and turned out many positive results, but there was no one studied the effect of Apigenin on DBCLB. On the one hand, as a kind of biotin extracted from plants, it is difficult for Apigenin to achieve its anti-cancer effect alone. In the other hand, small-molecule inhibitors have performed well in the treatment of a variety of tumors, but their effects are often limited due to its off-target side-effect or drug resistance. Therefore, we hypothesis that combined treatment of these two kinds of drugs may achieve more excellent and feasible anti-tumor effect. This assumption was indeed confirmed in our results, the combination of Apigenin and Abivertinib significantly inhibited the progression of subcutaneous tumors in mice injected with U2932. Unfortunately, we did not explore the survival time of these mice under the consideration that the life status of mice is not related to the size of the tumor, we believe that the survival time of mice is related to a variety of reasons and cannot explain the effect of drugs. In the study of the mechanism of inhibitory effect, our results *in vitro* experiments proved that the combination of the two drugs induced apoptosis which was also confirmed in the tunnel assay of the tumor moved from the mice.

In general, we believe that Apigenin can effectively inhibit proliferation of DBLCB cells and achieve better results in combination with Abivertinib, which may have some implications for clinical treatment of diffuse large B lymphoma.

## Supplementary Material

Supplementary figures.
