# Supplementary material for: Apigenin and Abivertinib, a novel BTK inhibitor synergize to inhibit diffuse large B-cell lymphoma in vivo and vitro
Source: J Cancer. 2020 Feb 3;11(8):2123–32. doi: 10.7150/jca.34981 (PMC7052937; doi:10.7150/jca.34981)
Supplement: Supplementary file 1 — Supplementary figures. [file jcav11p2123s1.pdf]

Apigenin and Abivertinib, a novel BTK inhibitor synergize to inhibit Diffuse large B-cell lymphoma *in vivo and vitro*

Shujuan Huang[1,2], Mengxia Yu[3] ,Nana Shi[4], Yile Zhou[1,2], Fengling Li[1,2], Xia Li[1,2] ,Xin Huang[1,2] , Jie Jin[1,2].

1 Department of Hematology, the First Affiliated Hospital, Zhejiang University College of Medicine, Hangzhou, People's Republic of China

2 Key Laboratory of Hematologic Malignancies, Diagnosis and Treatment, Zhejiang, Hangzhou, People's Republic of China

3 Department of Hematology, Hangzhou First people's hospital, Zhejiang, Hangzhou, China

4 The Children's Hospital Zhejiang University School of Medicine

Corresponding author: Prof. Jie Jin

Department of Hematology, the First Affiliated Hospital, Zhejiang University School of Medicine, No.79 Qingchun Road, Hangzhou 310003 Zhejiang, P.R. China.

E-mail: jiej0503@zju.edu.cn

Supplementary material

Fig.1

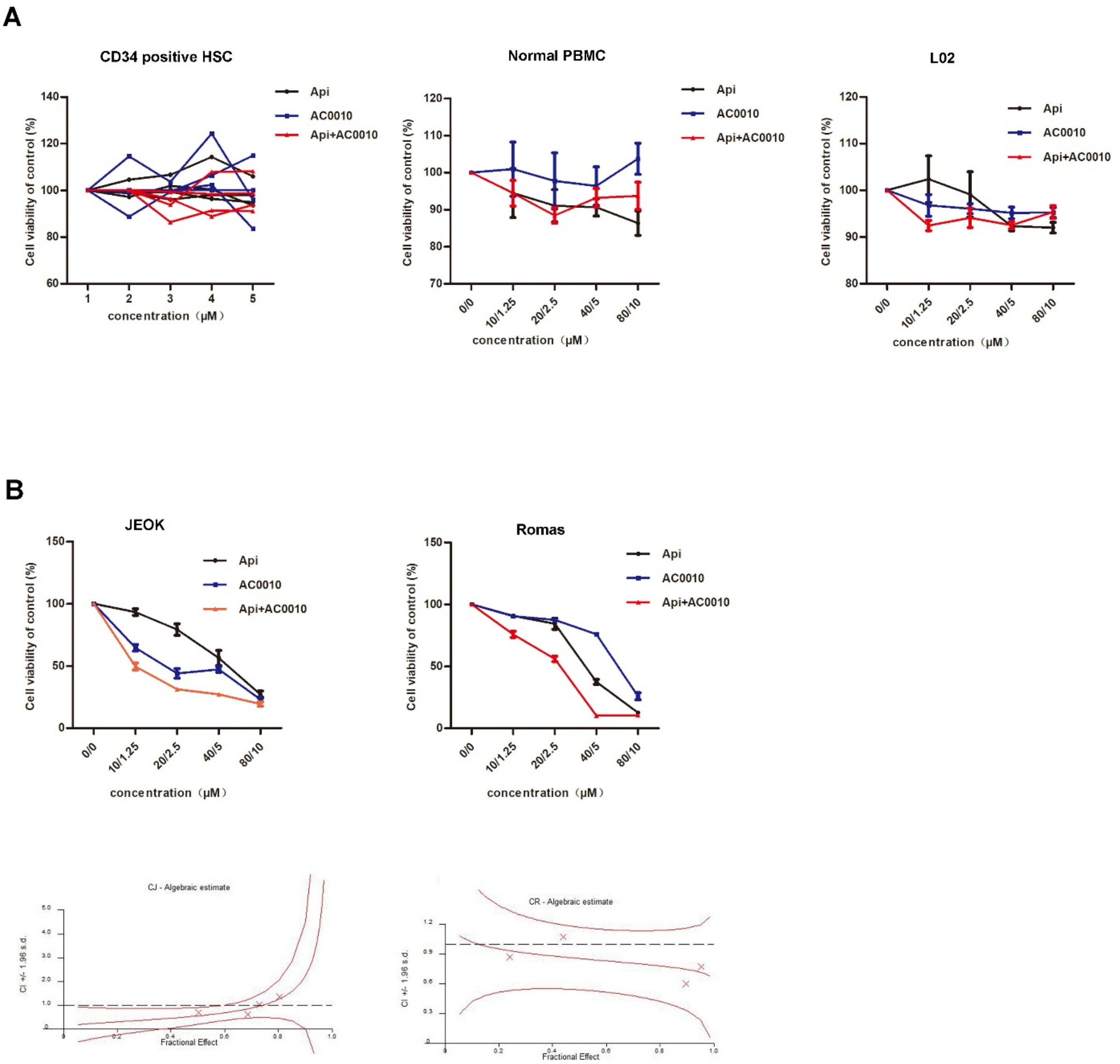

Fig.2

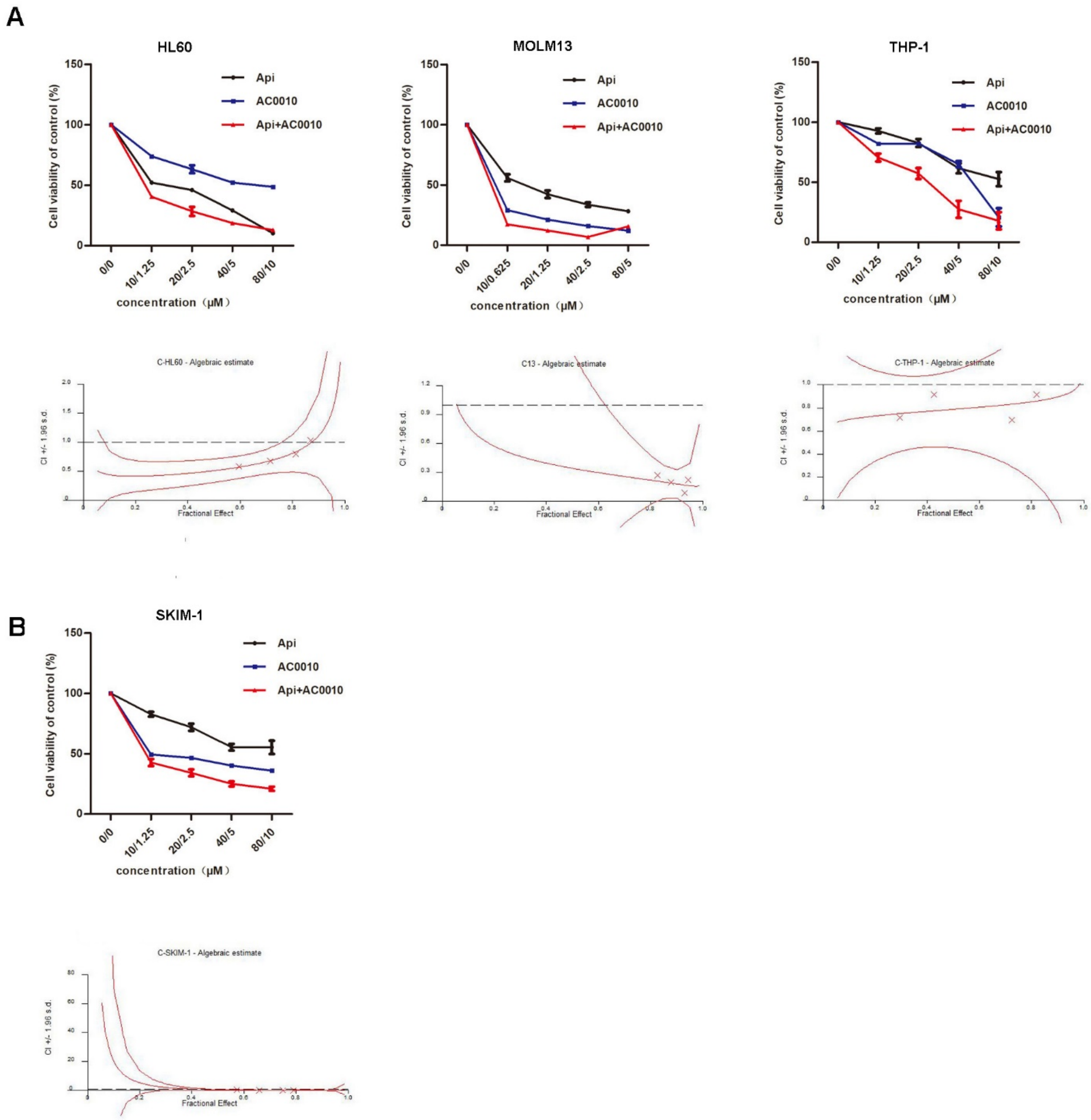

A
HL60
MOLM13
THP-1
Api
AC0010
Api+AC0010
Cell viability of control (%)
0/0
10/1.25
20/2.5
40/5
80/10
10/0.625
20/1.25
40/2.5
80/5
concentration（μM）
C-HL60 - Algebraic estimate
C13 - Algebraic estimate
C-THP-1 - Algebraic estimate
CI +/- 1.96 s.d.
Fractional Effect
B
SKIM-1
C-SKIM-1 - Algebraic estimate
